# Relationship between smoking and a new index of arterial stiffness, the cardio-ankle vascular index, in male workers: a cross-sectional study

**DOI:** 10.1186/1617-9625-10-11

**Published:** 2012-07-28

**Authors:** Koichi Hata, Toru Nakagawa, Mitsuhito Mizuno, Nobuaki Yanagi, Hiroko Kitamura, Takeshi Hayashi, Masataka Irokawa, Akira Ogami

**Affiliations:** 1 Department of Work Systems and Health, Institute of Industrial Ecological Science, University of Occupational and Environmental Health, 1-1 Iseigaoka, Kitakyusyu, 811-4341 Fukuoka, Japan; 2 Hitachi Health Care Center, Hitachi Ltd., 4-3-16 Osecho, Hitachi, 317-0076, Ibaraki, Japan

**Keywords:** Cardio-ankle vascular index, Smoking, Arterial stiffness, Cardiovascular disease

## Abstract

**Background:**

Cigarette smoking is one of the major factors that increases arterial stiffness. The purpose of this study was to examine further the relationship between smoking status and arterial stiffness using a new index, the cardio-ankle vascular index (CAVI), in male Japanese workers.

**Methods:**

This cross-sectional study included 4,729 male Japanese workers undergoing annual health checkups. CAVI was measured at the time of the annual health checkup between April 2007 and March 2008. The subjects were divided into three groups, smokers (n = 1,913), former smokers (n = 1,481) and non-smokers (n = 1,348) according to their responses to a questionnaire. We compared the CAVI in the three groups after adjusting for age. Multiple regression analysis was used to examine the association between CAVI and the number of cigarettes smoked per day in order to examine whether there was a dose–response relationship between smoking and CAVI.

**Results:**

The mean CAVI for each group was 7.81 ± 0.02 for smokers, 7.70 ± 0.02 for former smokers and 7.64 ± 0.02 for non-smokers. A significant difference was observed between each group. According to the results of multiple regression analysis, the standardized β of the number of cigarettes smoked per day was 0.09 (*p* < 0.01). This confirmed a positive association with CAVI.

**Conclusions:**

Our study demonstrated that there is a significant association between the number of cigarettes smoked per day and arterial stiffness, as measured by CAVI.

## Background

Cardiovascular disease is a major cause of death in industrialized countries [1]. In Japan, too, the rate of cardiovascular disease as a cause of morbidity has noticeably increased with the westernization of lifestyles. Arterial stiffening is a major factor in cardiovascular disease because of the reduced capacity in blood vessels and the concomitant rise in pulse pressure and fall in shear stress [2]. Therefore, assessment of arterial stiffness is believed to be useful in the prevention of cardiovascular disease.

Non-invasive indices of arteriosclerosis, especially brachio-ankle pulse wave velocity (baPWV), are commonly used for measuring arterial stiffness. An association has been demonstrated between baPWV values and arteriosclerotic diseases, including cardiovascular disease [3]. However, baPWV is strongly dependent on the subject’s blood pressure (BP) at the time of measurement and it is sometimes difficult to assess the baPWV value during treatment for hypertension or as a result of ‘white coat‘ hypertension. Recently, the cardio-ankle vascular index (CAVI), a new convenient method of non-invasive measurement, has been introduced to assess arterial stiffness. CAVI was developed by measuring PWV from the heart to the ankle, as well as BP [4]. CAVI represents arterial stiffness, specifically the elastic properties of the arterial wall between the aortic arch and distal arteries of the lower extremities. Several reports have shown that CAVI is associated with atherosclerotic disease and particularly reflects arteriosclerosis of the aorta, femoral and tibial arteries [4-6]. One of CAVI’s greatest merits is that measurements remain essentially unaffected by BP, in contrast to other non-invasive parameters of arterial stiffness [7].

Some studies in Japan suggested that chronic smoking affects arterial stiffness [8,9]. Cigarette smoking induces changes in both peripheral and central vascular function, even in young or middle aged smokers [10]. However, there are few large-scale studies on the relationship between smoking status and arterial stiffness in subjects of working age. In this cross-sectional study, we examined whether there was an association between smoking status and arterial stiffness, as measured by CAVI, and whether there was a dose–response relationship between smoking and CAVI.

## Methods

### Subjects and methods

Study subjects were participants in annual health examinations conducted between April 2007 and March 2008 by a Japanese electrical machinery manufacturing company who requested a CAVI assessment of arterial stiffness. CAVI examinations were performed in 4,831 male workers in a fasting state. Workers whose CAVI values were taken after a meal (n = 5) or were unreliable (six with ankle brachial index (ABI) <0.9, 65 with arrhythmia [4]) or could only be measured on one side (n = 23), as well as any workers who declined to provide information on their smoking status (n = 3), were excluded from our study. Finally, a total of 4,729 subjects were selected. Written informed consent was obtained from each examinee regarding the use of his data for research purposes prior to the study. This study was approved by the ethics committee of Hitachi General Hospital.

### Cardio-ankle vascular index

CAVI was measured by trained technicians using VaSera VS-1000 (Fukuda Denshi, Tokyo, Japan). Cuffs were placed bilaterally on the upper arms and ankles while the subjects were lying in a supine position with their heads held along the midline. Electrocardiography electrodes were placed on both wrists and a microphone was placed to detect heart sounds over the sternum. The participants rested in this supine position before monitoring commenced. The CAVI was calculated using the formula:

(1) $$\text{CAVI}=2\rho \times \text{ln}\;\left(\text{Ps}/\text{Pd}\right)\times \text{PWV}2/\Delta P$$

where Ps is systolic BP (SBP); Pd is diastolic BP (DBP);ΔP is SBP-DBP;ρis blood density; and PWV is pulse wave velocity. PWV was measured from the aortic valve to the ankle. Detailed CAVI measurement methods and principles have been previously reported [4]. All these measurements and calculations were performed automatically. The mean bilateral CAVI was used in our analysis. ABI was measured at the same time.

### Laboratory data

Height and weight were measured using an automated scale (Tanita BF-220, Tokyo, Japan) with the participants wearing a light gown. Body mass index (BMI) was calculated as the weight in kilograms divided by the square of height in meters. Other laboratory data obtained were glycosylated hemoglobin (HbA1c), blood sugar, total cholesterol, low-density lipoprotein (LDL) cholesterol, high-density lipoprotein (HDL) cholesterol and triglycerides in the fasting state.

### Blood pressure measurement

BP was measured by trained nurses with participants in the sitting position after sufficient rest. Pulse rate was measured at the same time. If SBP was over 140 mmHg or DBP was over 90 mmHg, BP was measured again. We used the ES-H55 (Terumo, Tokyo, Japan) to measure BP.

### Questionnaire

A self-administered questionnaire was used to collect information on smoking status (smoker, former smoker, non-smoker), the average number of cigarettes smoked per day (or previously smoked for former smokers), history of treatment for lifestyle diseases (diabetes mellitus, high BP, dyslipidemia) and average alcohol consumption per week. Average alcohol consumption per week was assessed by “go” in this study. A “go” is a traditional Japanese unit consisting of 23 g ethanol.

## Statistical analysis

Statistical analysis was performed using SPSS Version 15 for Windows (SPSS, Chicago, USA). Continuous and categorical data are expressed as mean ± standard deviation (SD) and number (percentage), respectively. A *p*-value < 0.05 was considered statistically significant. Baseline characteristics of the participants were compared by Tukey’s *t*-test. We compared the mean CAVI among the three groups: smokers, former smokers and non-smokers. However, non-invasive parameters of arterial stiffness, including CAVI, are strongly dependent on age. Therefore, data were compared as mean ± SD adjusted for age by analysis of covariance (ANCOVA). In addition, multiple stepwise regression analysis was performed to examine the relation between average number of cigarettes smoked per day and CAVI. Dependent variables were 1) age; 2) BMI; 3) average number of cigarettes smoked per day; 4) SBP; 5) DBP; 6) pulse rate; 7) LDL cholesterol; 8) HDL cholesterol; 9) triglycerides; 10) total cholesterol; 11) HbA1c; 12) alcohol consumption (the amount of “go” consumed per week).

## Results

The 4,729 subjects consisted of 1,907 smokers, 1,479 former smokers and 1,343 non-smokers. Baseline characteristics of the participants are shown in Table 1. The average ages of non-smokers, former smokers and smokers were 48.5 ± 8.6 years, 51.6 ± 7.5 years and 48.7 ± 8.1 years, respectively. Former smokers were significantly older than subjects belonging to the other two groups, but there was no significant difference between smokers and non-smokers (former smoker versus non-smoker: *p* < 0.01; former smoker versus smoker: *p* < 0.01; smoker versus non-smoker: *p* = 0.77). Non-smokers had significantly higher HDL-cholesterol (*p* < 0.01), SBP (*p* < 0.01) and DBP (*p* < 0.01) than smokers and significantly lower triglyceride (*p* < 0.01) and HbA1c (*p* < 0.01). Weekly alcohol consumption was significantly lower for non-smokers than for the other two groups (non-smoker versus former smoker: *p* < 0.01, non-smoker versus smoker: *p* < 0.01).

**Table 1:** Baseline characteristics of 4,729 male Japanese workers in this study between April 2007 and March 2008, according to Smoking Status

	**Non-smoker (n = 1,343)**	**Former smoker (n = 1,479)**	**Smoker (n = 1,907)**
Age (year)	48.5 ± 8.6	51.6 ± 7.5*	48.7 ± 8.1
Body Mass Index	24.3 ± 3.1*	24.3 ± 2.8*	24.0 ± 3.2
Blood pressure (mmHg)			
Systolic	121.5 ± 11.5*	122.7 ± 11.1*	118.9 ± 12.1
Diastolic	77.8 ± 8.0*	78.8 ± 7.6*	76.1 ± 8.3
Pulse rate (per minute)	68.9 ± 10.0	69.0 ± 9.8	68.2 ± 9.1
Fasting serum cholesterol (mg/dl)			
Low-density lipoprotein	126.0 ± 27.7	125.6 ± 27.7	124.6 ± 30.9
High-density lipoprotein	57.0 ± 13.7*	56.9 ± 14.3*	52.6 ± 13.1
Triglycerides	112.3 ± 1.6*	118.1 ± 1.7*	129.2 ± 1.7
Glucose (mg/dl)	104.3 ± 17.0	108.3 ± 22.7*	105.5 ± 21.5
Hemoglobin A1c (%)	5.4 ± 0.6*	5.5 ± 0.8	5.5 ± 0.8
Alcohol consumption (“go”/week)	3.86 ± 4.7*	6.11 ± 5.5*	5.91 ± 5.9
Number of cigarettes smoked per day		19.4 ± 8.5	19.0 ± 6.5
Under medical treatment (n (%))			
High blood pressure	150 (11.2 %)	269 (18.2 %)*	209 (11 %)
Dyslipidemia	86 (6.4 %)	155 (10.5 %)*	125 (6.6 %)
Diabetes mellitus	43 (3.2 %)*	95 (6.4 %)	95 (5 %)

Values are mean ± standard deviation or n (%).

The logarithmic mean of triglycerides was examined. High blood pressure was defined as > 140/90 mmHg (Japanese Society of Hypertension: Guidelines for the Management of Hypertension 2009).

Number of cigarettes smoked per day in former smoker: Number of cigarettes smoked per day when they were smokers.

*: *p- *value < 0.05 compared with smokers by Tukey's *t *-test or the Chi-square test.

**Figure 1:**
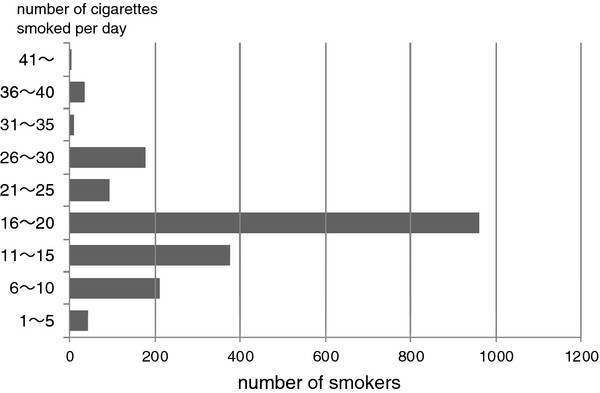
Smoking Frequency by number of cigarettes Smoked per day.

Figure1 shows the distribution of the average number of cigarettes smoked per day among smokers. The highest smoking frequency was an average number of 16 to 20 cigarettes per day. The frequency of more than 40 cigarettes per day was extremely low.

**Figure 2:**
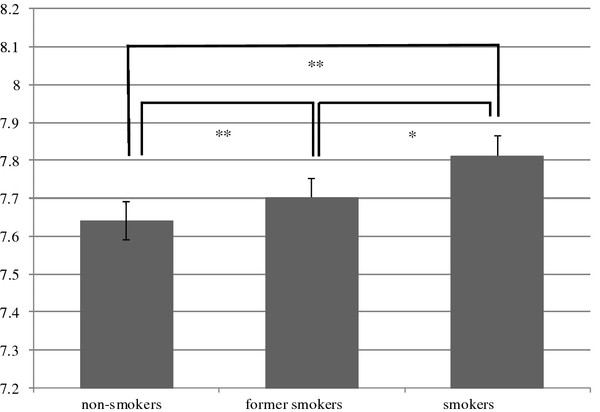
CAVI in Each Smoking Status Analysis of covariance (ANCOVA) was performed to adjust for age *: *P *value <0.01. Mean CAVI±SD of non-smokers, former smokers and smokers adjust by age are 7.64±0.02 and 7.81 ± 0.02 respectively.

The CAVI data among smokers, non-smokers and ex-smokers are presented in Figure2. We used ANCOVA to adjust for the influence of age. The average CAVI of all participants was 7.73. The CAVI of smokers, ex-smokers and non-smokers was 7.81 ± 0.02, 7.70 ± 0.02 and 7.64 ± 0.02, respectively. A significant difference was observed for each group (non-smoker versus former smoker: *p* < 0.01, non-smoker versus smoker: *p* < 0.01, former smoker versus smoker: *p* = 0.047).

Table 2 shows the result of stepwise multiple regression analysis between non-smokers and smokers. Age was the strongest factor that affected CAVI (β: 0.05, 95 % confidence interval (CI) of β: 0.05–0.056, *p* < 0.01). After adjustment for age, the average number of cigarettes smoked per day had the greatest influence on CAVI (β: 0.11, 95 % CI of β: 0.08-0.14, *p* < 0.01).

**Table 2:** Results of multiple linear regression analysis conducted to assess the relationship of CAVI to measures of variables in non-smoking group and smoking group (n = 3,250)

	***β***	**95%CI of *β***	**Standardized *β***	***p***
Age (actual age)	0.05	0.05–0.056	0.50	< 0.01
Number of cigarettes smoked per day (actual number)	0.11	0.08–0.14	0.09	< 0.01
R square			0.45	
Adjusted R square			0.45	

Dependent variables were 1) age; 2) body mass index; 3) average number of cigarettes smoked per day; 4) systolic blood pressure; 5) diastolic blood pressure; 6) pulse rate; 7) low-density lipoprotein cholesterol; 8) high-density lipoprotein cholesterol; 9) triglycerides; 10) total cholesterol; 11) HbA1c; 12) alcohol consumption (amount of “go” consumed per week). CI, confidence interval.

Stepwise procedures were used in this analysis.

## Discussion

The study indicated that CAVI, the new index of arterial stiffness, differs significantly after adjustment for age in Japanese male workers, depending on their smoking status. Many studies have reported that smoking status affects the non-invasive index of arterial stiffness or arteriosclerosis [8,9]. In addition, previous studies showed that the average number of cigarettes smoked per day influenced arterial stiffness [11]. We were able to confirm these relationships by using CAVI, which is the new index of arterial stiffness.

Various studies have also shown that smoking plays a major role in the occurrence of arteriosclerosis and arteriosclerotic disease [12-16]. Smoking promotes arteriosclerosis because it induces nitric oxide damage to vascular endothelial cells, contributes to high BP or diabetes mellitus, and stimulates coagulation and fibrinolysis pathways [17]. Arterial stiffness is thought to be associated with arteriosclerosis. However, it is ethically difficult to measure arteriosclerosis by invasive methods. CAVI measures arterial stiffness noninvasively and conveniently, and this advantage makes CAVI an appropriate method for assessment of arteriosclerosis caused by smoking in working people.

The CAVI of former smokers was lower than that of smokers but higher than that of non-smokers. Smoking cessation would not result in an immediate restoration of CAVI to non-smoker levels. It was shown that it took 10–14 years after smoking cessation for the majority of benefits to occur. There was no significant difference between the mortality rates of non-smokers and former smokers 10–14 years after smoking cessation [18]. Similarly, most other improvements in CAVI as a result of smoking cessation may take a long time.

The Brinkman index (the number of cigarettes smoked per day multiplied by the number of years of smoking) is commonly used to estimate the cumulative dose of smoking. In this study, however, we thought age would impact the Brinkman index and, therefore, we used the number of cigarettes smoked per day for our analysis.

Our study has several limitations. First, it is a cross-sectional study. A cohort study is required to accurately assess the causal correlation. We will continue to measure the subjects’ CAVI and obtain information on their smoking status at annual health checkups. Another limitation was the fact that all study subjects were male workers and thus our findings cannot be applied to women. There may be differences between men and women in CAVI due to lifestyle or disease and therefore it is necessary to conduct studies that include female subjects to determine any distinction between the genders.

## Conclusions

Our study showed an association between smoking status and CAVI in Japanese male workers. CAVI was higher in smokers than in non-smokers and ex-smokers adjusted for age. In addition, the number of cigarettes smoked per day is related to the degree of arterial stiffness, as measured by CAVI.

## Competing interests

The authors declare that they have no competing interests.

## Authors’ contributions

KH, TN and TH contributed to data collection. KH and TN contributed to data analysis. KH, MM, HK, and AK interpreted the data, and wrote the manuscript. All authors read and approved the final manuscript.
